# Performance of the Framingham coronary heart disease risk score for predicting 10-year cardiac risk in adult United Arab Emirates nationals without diabetes: a retrospective cohort study

**DOI:** 10.1186/s12875-020-01246-2

**Published:** 2020-08-26

**Authors:** Saif Al-Shamsi

**Affiliations:** Department of Internal Medicine, College of Medicine and Health Sciences, United Arab Emirates University, Al Ain, United Arab Emirates

**Keywords:** Coronary heart disease, Framingham, Risk prediction, United Arab Emirates, Validation

## Abstract

**Background:**

Primary prevention guidelines recommend the use of the Framingham risk score (FRS) to estimate the 10-year coronary heart disease (CHD) risk in patients without diabetes for statin eligibility. However, the FRS model has never been validated in an Arab population. Therefore, this study aimed to examine the clinical performance of the FRS model for predicting 10-year CHD risk in adult United Arab Emirates (UAE) nationals without diabetes.

**Methods:**

This 10-year retrospective cohort study included patients from the primary care clinics and outpatient specialty departments of a large tertiary care hospital in Al-Ain, UAE. They were aged 30–79 without a baseline history of cardiovascular disease and diabetes. The FRS for each subject was calculated. Follow-up data on hard CHD (hCHD) events (myocardial infarction or coronary death) for each participant were collected from the baseline visit in 2008 until December 31, 2019. The area under the time-dependent receiver operating characteristic (ROC) curve (AUROC) was used to assess the FRS model discrimination. Calibration was measured by using the Hosmer-Lemeshow χ^2^ test and the calibration curve. The optimal cutoff-point for hCHD risk prediction was determined by ROC curve analysis.

**Results:**

A total of 554 participants were included. The mean age was 48.0 ± 12.8 years and 45% were men. The mean predicted FRS of the study cohort was 5.2% and approximately 7% were classified as high-risk (≥ 20% threshold) by the FRS model. During a median follow-up of 10.2 years (interquartile range, 7.8–11.0 years), 26 hCHD events occurred. The FRS model displayed reasonably good discrimination (time-dependent AUROC value: 0.83) and calibration in predicting hCHD (Hosmer-Lemeshow χ^2^ statistic 11.2, *P* = 0.191). Applying the 20% high-risk threshold, the FRS model had a sensitivity of only 37% in identifying patients at high-risk for an hCHD event over 10 years. While a 7.5% optimal cutoff-point improved the sensitivity to 74%.

**Conclusions:**

The FRS can be used in the prediction of coronary risk among UAE nationals without diabetes, however, the recommended hCHD risk threshold for statin eligibility may be too high. Lowering the cutoff-point to 7.5% could improve the identification of patients for preventive treatment.

## Background

More than nine million deaths are attributed to coronary heart disease (CHD) globally according to a 2016 World Health Organization report [1]. In the Middle East, CHD is by far the most serious public health concern [2, 3]. Premature deaths associated with CHD have tripled over the past decade [4] and in a recent United Arab Emirates (UAE) study, the cumulative incidence of acute CHD events among men with one or more vascular risk factors was noted to be 8.9% over nine years [5]. While the crude incidence of acute CHD over ten years was 4.7% among European men in the general population [6]. This incidence is attributable to the high prevalence of risk factors for CHD in the UAE. When adjusted for age, the prevalence of smoking, diabetes, hypertension (HTN), and dyslipidemia is 11, 25, 29, and 51%, respectively [7]. Optimal, target-oriented primary prevention could help in reducing the incidence of CHD in the UAE population.

The morbidity and mortality associated with CHD and its related risk factors motivated research into the development of CHD risk assessment tools [8]. These tools, aid clinicians in the identification and risk stratification of individuals who would benefit from primary preventive strategies [9]. The Framingham risk score (FRS) [10], introduced in 2001, is recommended by the National Cholesterol Education Program-Adult Treatment Panel-III (NCEP-ATP-III) guidelines for the identification of high-risk individuals for lipid-lowering treatment [9]. The FRS model considers six traditional risk factors, age, sex, smoking, HTN, high-density lipoprotein-cholesterol (HDL-C), and total cholesterol (TC) to estimate a person’s absolute 10-year risk of incident CHD [8–10]. This simplified risk model was developed mainly among middle-aged Caucasian-Americans [8] and has been validated in different ethnic populations with varying results [10–12] although never in an Arab population.

Using unvalidated risk prediction tools could overestimate risk and result in administering inappropriate treatment, while underestimation could lead to undertreatment. Healthcare providers need to be confident in the accuracy of risk prediction models when applying them to the local population. Hence, this study sought to examine the clinical performance of the FRS model for predicting 10-year CHD risk in adult UAE nationals.

## Methods

### Study population and setting

**Fig. 1:**
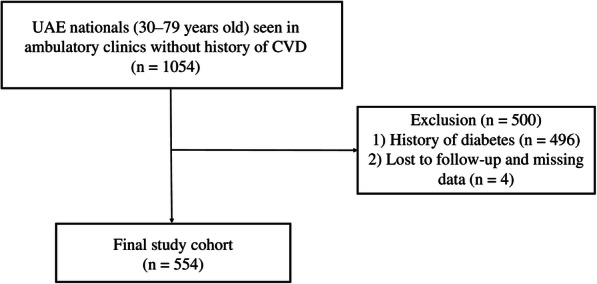
Cohort selection flow chart. UAE, United Arab Emirates; CVD, cardiovascular disease

Participants for this external validation study were selected from the primary care clinics and outpatient specialty departments of Tawam Hospital, a large government healthcare center, in Al-Ain, UAE. Electronic medical records (EMR) of 1054 Emirati participants aged 30–79 without a documented history of CHD, stroke, or peripheral arterial disease at baseline were retrospectively evaluated between April 1, 2008, and December 31, 2008. According to the NCEP-ATP-III guidelines, the FRS is not intended for patients with diabetes, which is considered as CHD risk equivalent [13, 14], therefore, 496 patients with diabetes and 4 additional participants who were lost to follow-up or with missing baseline data were excluded from the final analysis (Fig. 1).

Follow-up data on hard (hCHD) events, defined by the Framingham Heart Study as acute myocardial infarction, or coronary death [10], were collected through a review of the patient EMR from the 2008 baseline visit until December 31, 2019. The outcomes were confirmed according to clinicians’ diagnosis and death certificates. This study was approved by the Research Ethics Committees of Tawam Hospital and the UAE University (CRD 239/13).

### Predictor variable measurements

The baseline variables included age, sex, TC, HDL-C, systolic and diastolic blood pressure, the pharmacological treatment for HTN, and history of smoking (current or previous smokers). An automated oscillometric sphygmomanometer was used to measure blood pressure by qualified nurses.

Blood pressure, TC, and HDL-C in the Emirati validation cohort were categorized and compared along with age, sex, and smoking history to the Framingham study cohort [10]. HTN was classified based on the Seventh Joint National Committee on Hypertension guidelines: normal blood pressure and prehypertension, systolic ≤139 mmHg or diastolic ≤89 mmHg; stage 1 HTN, systolic 140–159 mmHg or diastolic 90–99 mmHg; stage 2 HTN, systolic ≥160 or diastolic ≥100 mmHg. The higher category was chosen if the systolic and diastolic blood pressures were in different categories [15]. Cutoff-points of TC (< 5.17 mmol/L, 5.17–6.18 mmol/L, ≥ 6.20 mmol/L) and HDL-C (≤ 1.53 mmol/L, ≥ 1.55 mmol/L) were used to categorize values according to the NCEP-ATP-III guidelines [9]. Lipid levels were analyzed by standard methods using a Beckman Coulter UniCel® DxC800 Synchron Clinical System.

### Statistical analysis

Differences in the baseline categorical variables between the Emirati validation and Framingham study cohorts were compared using Fisher’s exact test (two-sided). The 10-year predicted hCHD risk was calculated for each subject using the FRS model [10]. The predictor variables selected for the FRS model were age, sex, TC, HDL-C, systolic blood pressure, medications for HTN, and history of smoking. The calculated probabilities of risk were categorized into low (< 10%), intermediate (10–19.9%) and high (≥ 20%) risk [9] and stratified by observed hCHD events.

Discrimination and calibration were assessed to determine the predictive performance of the FRS model for 10-year hCHD risk in this validation cohort. Discrimination evaluates the model’s ability to differentiate individuals with and without the outcome event, based on the calculated predicted risk. The discriminatory power of the FRS model was evaluated by determining the area under the time-dependent receiver operating characteristic (ROC) curve (AUROC) at 10 years by using the Kaplan-Meier method [16]. Good discrimination was achieved if the time-dependent AUROC value was > 0.75. Calibration, which determines how closely the observed risk fit the predicted probabilities, was measured graphically and by using the Hosmer-Lemeshow χ^2^ test [17]. The calculated 10-year predicted hCHD risk was plotted against the actual incidence of hCHD events by deciles based on the predicted risk. A well-calibrated model would result in its calibration curve being closer to the 45-degree line, while a χ^2^ value < 20 indicates good calibration (*P* > 0.05).

The sensitivity, specificity, positive predictive value (PPV), and negative predictive value (NPV) were examined at 10 and 20% calculated FRS risk thresholds using the time-dependent ROC curve. The optimal high-risk cutoff-point for the study cohort was determined by applying the closest top left point approach to the ROC curve [18].

Statistical analyses were conducted using R software version 3.6.0 (The R Foundation for Statistical Computing, Vienna, Austria) and IBM SPSS software, version 26.0 (IBM Corporation, Armonk, NY, USA). A *P* value (two-tailed) less than 0.05 was considered statistically significant.

## Results

### Baseline characteristics

The baseline characteristics of the Emirati validation cohort and the original Framingham study cohort are displayed in Table 1. The mean age of the validation cohort was 48.0 ± 12.8 years and approximately 45% were men. Compared to the Framingham cohort, the prevalence of stage 2 HTN, smoking, diabetes, hypercholesteremia, and elevated HDL-C levels was lower in the Emirati subjects. The mean calculated 10-year FRS for incident hCHD of the Emirati validation cohort was 5.2%.

**Table 1:** Baseline predictors and outcomes in the Emirati validation and Framingham cohorts

Characteristic	Emirati validation cohort (***n*** = 554)	Framingham study cohort* (***n*** = 5251)	***P***-value†
Age (years), mean (SD)	48.0 (12.8)	49.0 (NA)	
Men, n (%)	249 (44.9)	2439 (46.4)	0.502
Blood pressure, n (%)			
Normal and prehypertension	408 (73.7)	3558 (67.7)	< 0.001
Stage 1 HTN	116 (20.9)	1095 (20.9)	
Stage 2 HTN	30 (5.4)	598 (11.4)	
TC, mmol/L, n (%)			
< 5.17	298 (53.8)	1996 (38.0)	< 0.001
5.17–6.18	166 (30.0)	1879 (35.8)	
≥ 6.20	90 (16.2)	1376 (26.2)	
HDL-C, mmol/L, n (%)			
≤ 1.53	482 (87.0)	3829 (72.9)	< 0.001
≥ 1.55	72 (13.0)	1422 (27.1)	
Smoking history, n (%)	102 (18.4)	2045 (38.9)	< 0.001
Diabetes mellitus, n (%)	0 (0.0)‡	234 (4.5)	< 0.001
Observed hCHD events, n (%)	26 (4.7)	273 (5.2)	

NA, not available; HTN, hypertension; TC, total cholesterol; HDL-C, high-density lipoprotein-cholesterol; hCHD, hard coronary heart disease; SD, standard deviation; FRS, Framingham risk score

*Data retrieved from D’Agostino et al. [10]

†*P*-values for categorical variables were calculated using Fisher’s exact test (2-tailed)

‡The FRS hCHD model is not intended for patients with diabetes, therefore excluded from the study

**Fig. 2:**
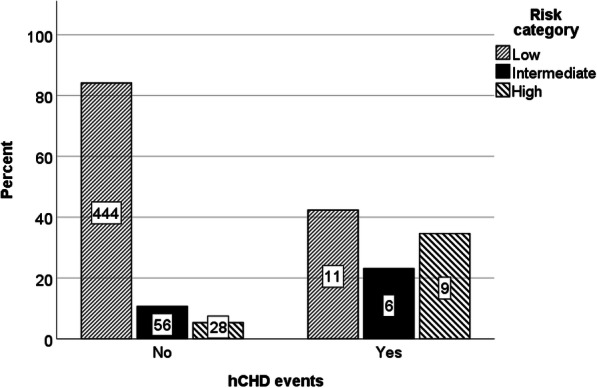
Comparison of risk categories of the FRS model and hCHD events observed. FRS, Framingham risk score; hCHD, hard coronary heart disease

The distribution of the risk categories of the FRS model and the actual hCHD events is shown in Fig. 2. More than 80% (455/554) of the Emirati validation cohort was classified as low risk by the FRS model.

### Predictive performance

In this Emirati validation cohort of 554 participants, 26 hCHD events occurred in total, during a median follow-up of 10.2 years (interquartile range, 7.8–11.0 years). Of the 26 individuals who experienced an hCHD event within 10 years, 42, 23, and 35% were categorized as low, intermediate, and high-risk, respectively, by the FRS model.

**Fig. 3:**
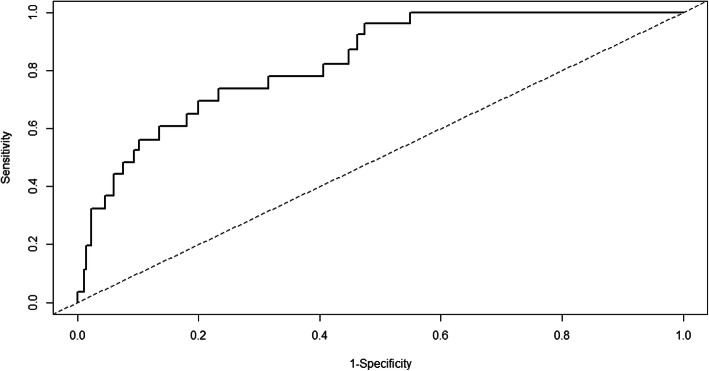
Time-dependent ROC curve analysis at 10 years using the FRS model in UAE nationals. ROC, receiver operating characteristic; FRS, Framingham risk score; UAE, United Arab Emirates

**Fig. 4:**
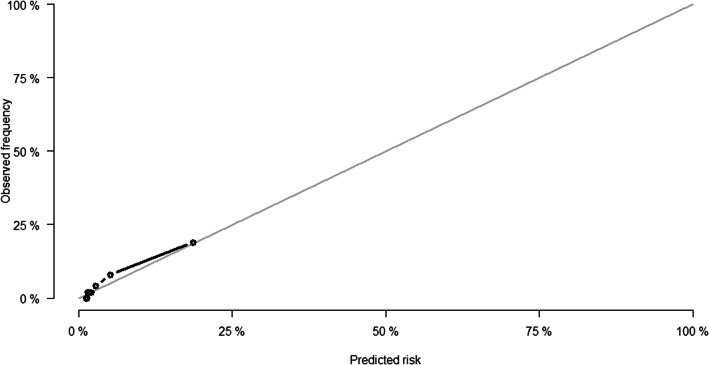
Calibration plots of observed and predicted 10-year CHD events in UAE nationals using the FRS. CHD, coronary heart disease; UAE, United Arab Emirates; FRS, Framingham risk score

The time-dependent AUROC for the FRS model at 10 years was 0.83, standard error (SE) 0.04, (Fig. 3), indicating a good discriminatory ability to differentiate hCHD events from non-events. The calibration curve in Fig. 4 demonstrated, overall, good agreement between the observed risk and predicted risk. The Hosmer-Lemeshow χ^2^ statistic was 11.2 (*P* = 0.191).

### Sensitivity and specificity

Table 2 depicts the time-dependent sensitivity, specificity, PPV, and NPV of the NCEP-ATP-III recommended high-risk threshold of 20% for hCHD 10-year risk and the 7.5% optimal cutoff-point determined by ROC curve analysis. Sensitivity at the 20% risk threshold and 7.5% optimal cutoff-point was 37 and 74%, respectively, while specificity was 94 and 77%, respectively.

**Table 2:** Sensitivity, specificity, PPV, and NPV of the FRS model for the prediction of 10-year hCHD

Cut-off predicted risk threshold, %	Sensitivity, % (SE)	Specificity, % (SE)	PPV, % (SE)	NPV, % (SE)
≥ 20	36.9 (9.9)	94.4 (1.4)	25.2 (7.9)	96.7 (0.8)
≥ 10	60.9 (10.1)	82.7 (2.3)	15.3 (3.8)	97.6 (0.8)
≥ 7.5*	73.8 (9.2)	76.7 (2.6)	14.0 (3.2)	98.3 (0.7)

PPV, positive predictive value; NPV, negative predictive value; FRS, Framingham risk score; hCHD, hard coronary heart disease; SE, standard error; ROC, receiver operating characteristic

*Optimal cutoff-point determined by ROC curve analysis

## Discussion

This validation study examined the clinical performance of the FRS model in an external Emirati cohort. This study found the FRS model to have both reasonably good discrimination and calibration in predicting hCHD among UAE nationals without diabetes. However, the sensitivity of the recommended decision threshold of 20% for identifying high-risk individuals was relatively poor.

Since the NCEP-ATP-III guideline for cholesterol management was released in 2001, several studies have researched the generalizability of the FRS model for predicting 10-year hCHD risk in different ethnic populations. In a study that assessed the applicability of the FRS equation to non-Caucasian Americans, found that although the model discriminated well in Native Americans, Hispanic, and Japanese Americans, it overestimated their risk, particularly in higher predicted risk categories [10]. Similarly, in studies of Asian populations, the FRS was observed to have moderate discrimination and poor calibration, with greater variations noted in the higher deciles of risk [11, 19].

So far, there is no published literature on the performance of prediction models in an Arab population. Although this study provides the first evidence that the FRS (NCEP-ATP-III) model accurately predicts coronary risk in UAE nationals without diabetes, the recommended 20% high-risk threshold may be excessive. With this threshold, the FRS model correctly identified only approximately 37% of patients who subsequently experienced an hCHD event within 10 years as being at high-risk (sensitivity). A study conducted in India that included 740 CHD patients without diabetes, found that the FRS identified only 15.1% of these patients as high-risk [20]. This disproportionate underestimation of risk by the FRS model, when using the recommended risk thresholds, may lead to patients who need it most being deprived of the necessary preventive treatment. However, according to the current study’s ROC curve analysis, selecting the optimal cutoff-point of 7.5% could potentially correctly identify about 74% of UAE nationals who would experience an hCHD event within 10 years.

The results of this study may have clinical implications for CHD prevention in UAE nationals without diabetes. Cardiovascular risk prediction tools, such as the FRS model, have been widely recommended by international and local guidelines to better target primary preventive treatments, particularly lipid-lowering therapy, in susceptible individuals [15, 21]. However, the FRS model does not include emerging risk factors, such as renal failure, which is associated with increased cardiovascular risk among UAE nationals [5]. Therefore, developing novel risk prediction models based on local data would be more appropriate in populations with varying disease patterns and risk factors.

### Strengths and limitations

This study’s 10-year follow-up period and the criteria used to define predictor variables and hCHD events align with the original Framingham study. However, several limitations need to be considered. First, this study used ambulatory patients’ data derived from the EMR of a single large tertiary care center, therefore, the generalizability of the results to the broader UAE population may be limited. However, the overall mean 10-year cardiac risk of 5.2% estimated at baseline is comparable to the results of several large population-based studies in the UAE [21, 22]. Second, the original derivation cohort from the Framingham study did not include individuals aged < 30 years, therefore the FRS tool may be imprecise in this population group. Finally, selection bias may have occurred, on account of the study’s retrospective cohort design.

## Conclusion

In summary, this study demonstrated the extendibility of the FRS (NCEP-ATP-III) model in the prediction of coronary risk in UAE nationals without diabetes. However, the recommended hCHD risk threshold of 20% for lipid-lowering initiation may be too high and could lead to undertreatment. In the absence of a locally developed cardiac risk prediction tool, the FRS model could be used by primary care providers. Lowering the hCHD high-risk threshold to 7.5% could improve the risk-stratification of patients for preventive treatment in the UAE.

## Data Availability

The data that support the findings of this study are available from the corresponding author upon reasonable request.

## Abbreviations

AUROC: Area under the receiver operating characteristic
CHD: Coronary heart disease
EMR: Electronic medical records
FRS: Framingham risk score
hCHD: Hard coronary heart disease
HDL-C: High-density lipoprotein-cholesterol
HTN: Hypertension
NCEP-ATP-III: National Cholesterol Education Program-Adult Treatment Panel-III
NPV: Negative predictive value
PPV: Positive predictive value
ROC: Receiver operating characteristic
TC: Total cholesterol
UAE: United Arab Emirates

## References

[1] Global Health Estimates 2016: Deaths by Cause, Age, Sex, by Country and by Region, 2000–2016. Geneva: World Health Organization; 2018. World Health Organization. http://www.who.int/healthinfo/global_burden_disease/estimates/en/. Accessed 17 Mar 2020.

[2] Aljefree N, Ahmed F (2015). Prevalence of cardiovascular disease and associated risk factors among adult population in the Gulf region: a systematic review. Adv Public Health.

[3] Motlagh B, O’Donnell M, Yusuf S (2009). Prevalence of cardiovascular risk factors in the Middle East: a systematic review. Eur J Cardiovasc Prev Rehabil.

[4] United Arab Emirates. Institute for Health Metrics and Evaluation. 2015. http://www.healthdata.org/united-arab-emirates. Accessed 17 Mar 2020.

[5] Al-Shamsi S, Regmi D, Govender RD (2019). Incidence of cardiovascular disease and its associated risk factors in at-risk men and women in the United Arab Emirates: a 9-year retrospective cohort study. BMC Cardiovasc Disord.

[6] Menotti A, Lanti M, Puddu PE, Kromhout D (2000). Coronary heart disease incidence in northern and southern European populations: a reanalysis of the seven countries study for a European coronary risk chart. Heart Br Card Soc.

[7] Hajat C, Harrison O, Al SZ (2012). Weqaya: a population-wide cardiovascular screening program in Abu Dhabi, United Arab Emirates. Am J Public Health.

[8] Wilson PW, D’Agostino RB, Levy D, Belanger AM, Silbershatz H, Kannel WB (1998). Prediction of coronary heart disease using risk factor categories. Circulation..

[9] Expert Panel on Detection, Evaluation, and Treatment of High Blood Cholesterol in Adults. Executive summary of The Third Report of The National Cholesterol Education Program (NCEP) Expert Panel on Detection, Evaluation, And Treatment of High Blood Cholesterol In Adults (Adult Treatment Panel III). JAMA. 2001;285:2486–2497. 10.1001/jama.285.19.2486 11368702

[10] D’Agostino RB (2001). Grundy S, Sullivan LM, Wilson P, CHD risk prediction group. Validation of the Framingham coronary heart disease prediction scores: results of a multiple ethnic groups investigation. JAMA..

[11] Liu J (2004). Predictive value for the Chinese population of the Framingham CHD risk assessment tool compared with the Chinese multi-provincial cohort study. JAMA..

[12] Brindle P, Emberson J, Lampe F, Walker M, Whincup P, Fahey T (2003). Predictive accuracy of the Framingham coronary risk score in British men: prospective cohort study. BMJ..

[13] Coleman RL, Stevens RJ, Retnakaran R, Holman RR (2007). Framingham, SCORE, and DECODE risk equations do not provide reliable cardiovascular risk estimates in type 2 diabetes. Diabetes Care.

[14] Framingham Heart Study. https://framinghamheartstudy.org/fhs-risk-functions/hard-coronary-heart-disease-10-year-risk/. Accessed 17 Mar 2020.

[15] National High Blood Pressure Education Program. The Seventh Report of the Joint National Committee on Prevention, Detection, Evaluation, and Treatment of High Blood Pressure. Bethesda (MD): National Heart, Lung, and Blood Institute (US); 2004. http://www.ncbi.nlm.nih.gov/books/NBK9630/. Accessed on date 17 Mar 2020. 20821851

[16] Heagerty PJ, Lumley T, Pepe MS (2000). Time-dependent ROC curves for censored survival data and a diagnostic marker. Biometrics..

[17] Lemeshow S, Hosmer DW (1982). A review of goodness of fit statistics for use in the development of logistic regression models. Am J Epidemiol.

[18] Perkins NJ, Schisterman EF (2006). The inconsistency of “optimal” cutpoints obtained using two criteria based on the receiver operating characteristic curve. Am J Epidemiol.

[19] Asia Pacific Cohort Studies Collaboration (2007). Cardiovascular risk prediction tools for populations in Asia. J Epidemiol Community Health.

[20] Garg N, Muduli SK, Kapoor A, Tewari S, Kumar S, Khanna R (2017). Comparison of different cardiovascular risk score calculators for cardiovascular risk prediction and guideline recommended statin uses. Indian Heart J.

[21] Hajat C, Harrison O (2010). The Abu Dhabi cardiovascular Program: the continuation of Framingham. Prog Cardiovasc Dis.

[22] Yusufali A, Bazargani N, Muhammed K, Gabroun A, AlMazrooei A, Agrawal A (2015). Opportunistic screening for CVD risk factors: the Dubai shopping for cardiovascular risk study (DISCOVERY). Glob Heart.

